# Inhibition of histone deacetylase 6 suppresses inflammatory responses and invasiveness of fibroblast-like-synoviocytes in inflammatory arthritis

**DOI:** 10.1186/s13075-021-02561-4

**Published:** 2021-07-05

**Authors:** Jin Kyun Park, Sehui Shon, Hyun Jung Yoo, Dong-Hyeon Suh, Daekwon Bae, Jieun Shin, Jae Hyun Jun, Nina Ha, Hyeseung Song, Young Il Choi, Thomas Pap, Yeong Wook Song

**Affiliations:** 1 Division of Rheumatology, Department of Internal Medicine, Seoul National University College of Medicine, Seoul, South Korea; 2 Department of Molecular Medicine and Biopharmaceutical Sciences, Graduate School of Convergence Science and Technology, Seoul National University, Seoul, South Korea; 3 CKD Research Institute, Yongin-si, Gyeonggido South Korea; 4 Division of Mol Medicine of Musculoskeletal Tissue, University Munster, Munster, Germany; 5 Medical Research Center, Institute of Human-Environment Interface Biology, Seoul National University, Seoul, South Korea

**Keywords:** Histone deacetylase 6, Inhibitor, Rheumatoid arthritis, Inflammation, Fibroblast-like synoviocytes

## Abstract

**Background:**

To investigate the effects of inhibiting histone deacetylase (HDAC) 6 on inflammatory responses and tissue-destructive functions of fibroblast-like synoviocytes (FLS) in rheumatoid arthritis (RA).

**Methods:**

FLS from RA patients were activated with interleukin (IL)-1β in the presence of increasing concentrations of M808, a novel specific HDAC6 inhibitor. Production of ILs, chemokines, and metalloproteinases (MMPs) was measured in ELISAs. Acetylation of tubulin and expression of ICAM-1 and VCAM-1 were assessed by Western blotting. Wound healing and adhesion assays were performed. Cytoskeletal organization was visualized by immunofluorescence. Finally, the impact of HDAC6 inhibition on the severity of arthritis and joint histology was examined in a murine model of adjuvant-induced arthritis (AIA).

**Results:**

HDAC6 was selectively inhibited by M808. The HDAC6 inhibitor suppressed the production of MMP-1, MMP-3, IL-6, CCL2, CXCL8, and CXCL10 by RA-FLS in response to IL-1β. Increased acetylation of tubulin was associated with decreased migration of RA-FLS. Inhibiting HDAC6 induced cytoskeletal reorganization in RA-FLS by suppressing the formation of invadopodia following activation with IL-1β. In addition, M808 tended to decrease the expression of ICAM-1 and VCAM-1. In the AIA arthritis model, M808 improved the clinical arthritis score in a dose-dependent manner. Also, HDAC6 inhibition was associated with less severe synovial inflammation and joint destruction.

**Conclusion:**

Inhibiting HDAC6 dampens the inflammatory and destructive activity of RA-FLS and reduces the severity of arthritis. Thus, targeting HDAC6 has therapeutic potential.

## Background

Rheumatoid arthritis (RA) is a systemic autoimmune disease characterized by chronic synovial inflammation, which leads to progressive damage to cartilage and bone and, eventually, to joint destruction [1]. Synovial inflammation, or synovitis, is caused by continuous recruitment and activation of immune cells within the synovial membranes of the joints [2]. Infiltrating immune cells interact with synoviocytes, which outline the synovial membrane, and transform them into fibroblast-like synoviocytes (FLS). FLS secrete proinflammatory cytokines, including tumor necrosis factor (TNF)-α and interleukin (IL)-1, IL-6, and tissue-degrading metalloproteinases (MMPs), into the joint spaces of RA patients [3]. More importantly, RA-FLS migrate and invade the adjacent bone tissue (much like invading cancer cells), with subsequent bone erosion and joint destruction [4, 5]. Therefore, inhibiting FLS-mediated proinflammatory responses and subsequent tissue destruction might be a novel therapeutic target. A potential approach could be to alter post-translational modification (PTM) of cytoskeletal proteins in FLS by regulating histone deacetylase (HDAC) [6, 7]. HDAC removes acetyl-groups from core histones in DNA, thereby repressing gene transcription by condensing the DNA structure [8–10]. Among HDACs, HDAC6 is located primarily in the cytoplasm, where it deacetylates non-histone proteins such as α-tubulin and cortactin [11, 12]. This post-translational protein modification impacts a variety of cellular processes/functions, including intracellular transport, cell migration and proliferation, and cell-to-cell interactions [13]. HDAC6 activity is upregulated in the synovial tissues of RA patients [14]. Overexpression of HDAC6 in tissue-resident macrophages leads to spontaneous production of inflammatory cytokines [15]. Interestingly, HDAC6 activity regulates tissue infiltration of fibroblasts in breast cancer, suggesting a critical role in tissue invasiveness [10, 16]. Accordingly, overexpression of HDAC6 might promote invasiveness of FLS; therefore, a HDAC6 inhibitor might prevent cancer cell-like tissue invasion and joint destruction by RA-FLS [17, 18].

We and others showed that HDAC6 inhibitors suppress the inflammatory response of immune cells and ameliorate arthritis in a murine RA model [19, 20]. However, the impact of HDAC6 inhibition on RA-FLS function remains unclear. M808 (hydroxamic acid) is a potent and selective HDAC6 inhibitor with better selectivity and solubility than the structurally related compound ACY-1215 [21].

Here, we examined whether inhibiting HDAC6 with M808 suppresses inflammatory responses of RA-FLS and ameliorates inflammation and joint destruction in vivo.

## Methods

### Enzyme kinetic assay

To evaluate the potency and selectivity of M808, a novel HDAC6 inhibitor, an HDAC panel assay was carried out by Reaction biology Corp. (PA, USA). Fluorogenic peptides from p53 residues 379–382 (RHKK(Ac)AMC) were used as substrates for HDAC 1, 2, 3, 6, and 10; a fluorogenic HDAC class 2a substrate (trifluoroacetyl lysine) was used for HDAC 4, 5, 7, 9 and 11; and a fluorogenic peptide (p53 residues 379–382 (RHK(Ac)K(Ac)AMC)) was used for HDAC8. HDAC isotypes were incubated with these substrates in the presence of M808 in a reaction buffer comprising 50 mM Tris-HCL (pH 8.0), 137 mM NaCl, 2.7 mM KCl, 1 mM MgCl_2_, and 1 mg/mL bovine serum albumin. After adding an equal volume of trypsin/TSA solution, fluorescence was detected at an excitation wavelength of 360 nm and an emission wavelength of 460 nm_._

For kinetic studies of M808, 0.042 μM HDAC6 (#382180, Calbiochem) was mixed with increasing concentrations of M808 (0, 1.52, 4.57, 13.72, and 41.15 nM), followed by addition of 1.56, 3.12, 6.25, 12.5, 25, or 50 μM RHKK-AC-AMC (Enzo Life Science). The deacetylation reaction was stopped by adding trypsin/TSA solution. After 20 min, AMC release was measured in a microplate reader (Envision, PerkinElmer) at λ_Ex_360 nm/λ_Em_460 nm. Kinetics were calculated from a Michaelis-Menten plot (V = Vmax[S]/(Km + [S])) and a double reciprocal plot or Lineweaver-Burk plot (1/V = (Km + [S])/Vmax[S] = (Km/Vmax)(1/[S]) + (1/Vmax)) using the GraphPad Prism 4.0 program. Other kinetic parameters were calculated as described by Wegener et al. [22]. V is the reaction velocity or reaction rate, Km is the Michaelis-Menten constant, Vmax is the maximum action velocity, and [S] is the substrate (M808) concentration.

### Patients

Patients who fulfilled the 1987 American College of Rheumatology (ACR) criteria for the classification of RA were enrolled after obtaining informed consent [23]. The study was approved by the Institutional Review Board (IRB No. 1603-141-751).

### FLS preparation

Synovial tissue was obtained from RA patients during knee joint replacement surgery. The tissue was minced and then treated with type II collagenase (Worthington, OH, USA) at 37 °C. FLS were obtained by filtration through a 40-μm cell strainer and then allowed to adhere to a culture plate. After removing non-adherent cells, FLS were cultured in DMEM supplemented with 10% fetal bovine serum and 1% penicillin/streptomycin (P/S). FLS from the fourth to the eighth passage were used for experiments.

### Cytokines, chemokines, and MMP assay

FLS were pre-treated for 1 h with increasing concentrations of the HDAC6 inhibitor and then stimulated for 24–72 h with 10 ng/mL IL-1β (R&D systems, MN, USA).

The culture supernatants were collected and IL-6 concentrations were measured in a Human IL-6 DuoSet enzyme-linked immunosorbent assay (ELISA) (R&D systems). MMP-1, MMP-3, CCL2, CXCL8, and CXCL10 concentrations were measured using Magnetic Luminex Screening Assay multiplex kits (Luminex assay, R&D systems).

### Western blot analysis

RA-FLS were treated with 10 ng/mL IL-1β in the presence of tubastatin A and M808 for 24 h. Protein lysates of FLS were separated in SDS-PAGE gels and transferred to nitrocellulose membranes. Membranes were incubated overnight at 4 °C with primary antibodies specific for HDAC6 (Cell Signaling Technology, MA, USA), acetyl-tubulin, α-tubulin (Sigma-Aldrich), ICAM-1, VCAM-1, or GAPDH (Santa Cruz, TX, USA). After washing, the membranes were incubated for 2 h at room temperature with the HRP-conjugated anti-mouse or anti-rabbit antibodies (Jackson Immuno Research Laboratories, PA, USA). Target proteins were detected using a chemiluminescent substrate (Elpis Biotech, South Korea).

### Cell lines

U937 cells (ATCC CRL-1593.2), a human monocyte cell line, were purchased from ATCC. Jurkat cells, an immortalized line of human T lymphocytes, were purchased from Korean Cell Line Bank. The cell lines were cultured in RPMI-1640 (GIBCO Invitrogen, Basel, Switzerland) containing 10% fetal bovine serum (FBS) and 1% penicillin/streptomycin until use.

### Adhesion assay

U937 and Jurkat cells were labeled with 5 μM calcein-AM (Molecular Probes, Thermo Fisher Scientific) at 37 °C for 30 min. The cells were washed with serum-free RPMI and resuspended at a density of 1 × 10^6^ cells/mL. RA-FLS were treated with the HDAC inhibitor at 37 °C for 1 h in the presence of U937 cells or Jurkat cells. Unattached cells were removed by washing with phosphate-buffered saline (PBS) and adherent cells were lysed with 0.1% Triton X-100 (Sigma-Aldrich) in PBS. The fluorescence of the calcein-labeled U937 and Jurkat cells adhering to RA-FLS were analyzed in a SpectraMax M5 device (Molecular Devices) at an excitation wavelength of 494 nm and an emission wavelength of 517 nm.

### Cell migration assay

The effect of HDAC6 inhibition on the migration of RA-FLS was determined by a wound healing assay. RA-FLS were seeded onto a culture dish until a confluence of monolayer was observed. The RA-FLS monolayer was scratched (i.e., wounded) with a sterile pipette tip to create an artificial homogenous wound of a cell-free area. The cells were then stimulated with IL-1β with or without pretreatment with the HDAC6 inhibitor. Images of the cells migrating into the wound were obtained every 24 h for 72 h under a light microscope (DFC295, Leica, Wetzlar, Germany) and assessed using LAS V3.8 (Leica) software. The number of cells migrating into the wound was counted.

### Immunofluorescence staining

FLS were seeded onto 8-chamber slides (Becton Dickinson and Company, Franklin Lakes, NJ) and then treated with IL-1β and M808. After treatment, the cells were fixed for 10 min in 4% paraformaldehyde and permeabilized for 20 min with PBS containing 1% Triton X-100 (Sigma-Aldrich, St. Louis, MO). The cells were then stained with an Alexa Fluor 488-labeled anti-phalloidin antibody (Cell Signaling Technology, Beverly, MA) and an anti-cortactin antibody (Cell Signaling Technology) that was detected with an Alexa Fluor 568-labeled secondary antibody (Cell Signaling Technology, Beverly, MA). Nuclei were counterstained for 5 min with DAPI (Thermo Fisher Scientific, Waltham, MA). Thereafter, cells were mounted in ProLong^TM^ Gold antifade reagent (CST) and examined under an inverted fluorescence microscope (EVOS FL Cell Imaging System; Life Technologies, Darmstadt, Germany).

### Experimental murine arthritis model

Female Lewis rats (approximately 5 weeks old) were purchased from Central Lab Animal, Inc. (Seoul, Republic of Korea). Animals were divided into six groups (eight or nine animals per group). Each group received oral M808 (0, 3, 10, 30, 50, and 100 mg/kg) once a day, starting 1 day (D-1) before injection of complete Freund’s adjuvant (CFA, Chondrex, WA, USA). To induce arthritis, each animal received an injection of 100 μL CFA into the tail base. The clinical score of arthritis and body weight were measured on days 9, 13, and 16 post-CFA injection. Rats were sacrificed on day 16.

### Arthritis assessment

The severity of arthritis was evaluated by calculating a clinical score for each joint; this was based on joint swelling and erythema (scored 0 to 4). The clinical scores for four joints were summed to obtain a total score for each animal, as described previously [19].

For histological evaluation, joint sections of the hind paw (including tarsal bones, metatarsal bones, and digits) were obtained and stained with hematoxylin and eosin. Synovitis was scored on a scale of 0–4 as follows: 0 = normal; 1 = mild synovial hypertrophy (< 5 cell layers) with few inflammatory cells; 2 = moderate synovial hypertrophy (< 20 cell layers) with inflammatory infiltrates; 3 = pannus, fibrous tissue, abscess, and interstitial edema; and 4 = pannus and fibrous tissue formation, abscess, and interstitial edema on both sides of a joint. Cartilage damage was scored as follows: 0 = normal, 1 = minor (< 10%), 2 = moderate (10~50%), 3 = severe (50~80%), and 4 = very severe (80~100%) damage of the cartilage. Bone erosion was scored as follows: 0 = normal, 1 = mild (focal subchondral erosion), 2 = moderate (multiple subchondral erosions), 3 = severe (multiple subchondral erosions and focal erosion), and 4 = very severe (multiple erosions in multiple joints). Investigators, all of whom were blinded to treatment, evaluated joint histology.

### Statistical analysis

Data are expressed as the mean ± SEM. The results of the experiments were compared using t-tests. A p value < 0.05 was deemed statistically significant. Statistical analyses were performed using GraphPad Prism 5 (GraphPad, CA, USA).

## Results

### M808 is a novel selective HDAC6 inhibitor

**Fig. 1:**
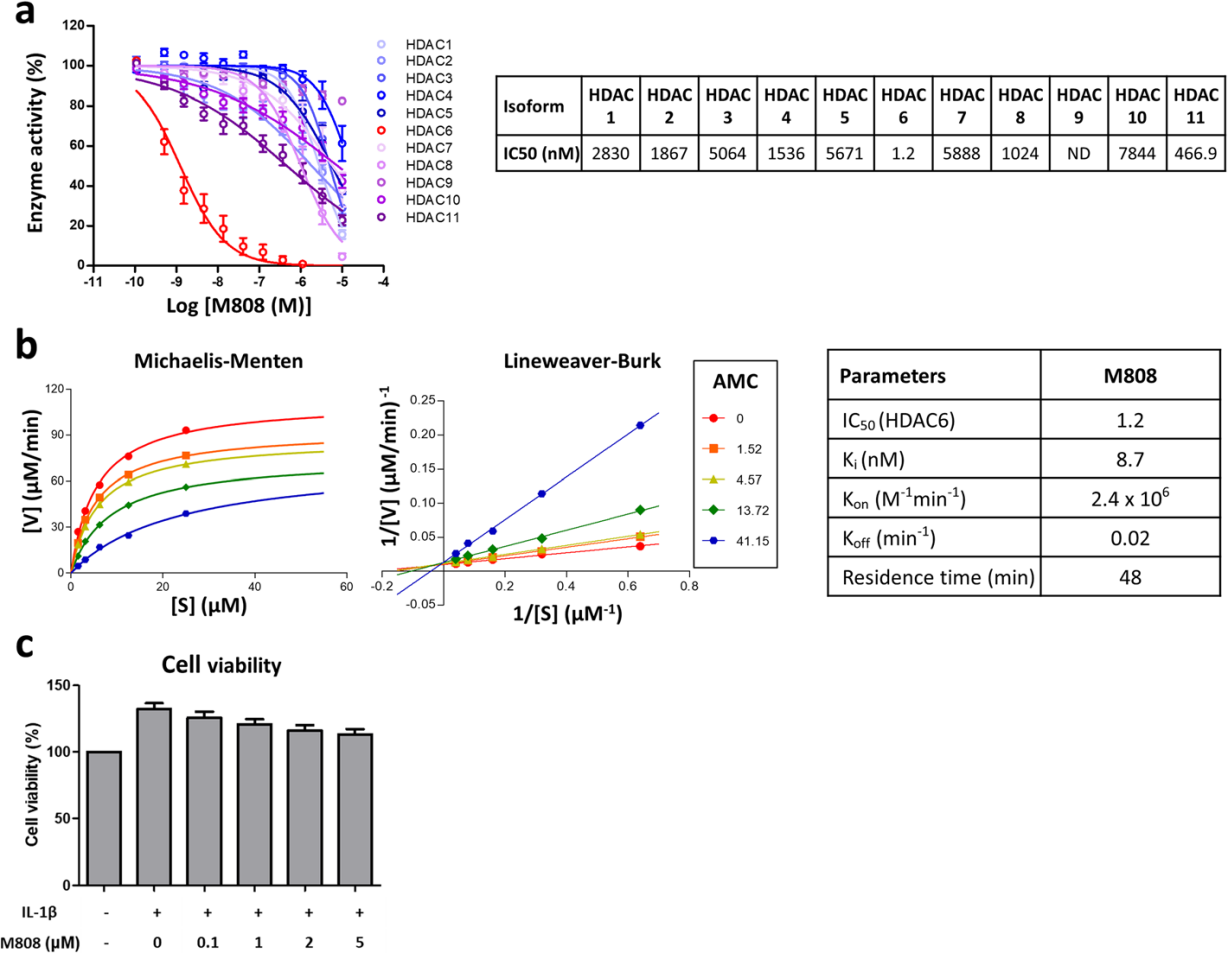
M808 is a selective HDAC6 inhibitor. **a** Selectivity of M808 for HDAC6. Inhibition of HDAC is dependent on the M808 concentration (IC50 values are shown). **b** Kinetics of M808 were determined by measuring the activity of HDAC6 in the presence of M808. **c** Cells were incubated with IL-1β and increasing concentrations of M808. Cell viability was then measured. AMC, RHKK-AC-AMC; IC50, the half maximal inhibitory concentration; IL, interleukin; Ki, inhibitory constant; ND, not determined; S, substrate; V, velocity

First, we determined the biochemical selectivity of the novel HDAC6 inhibitor M808. The half maximal inhibitory concentration IC50 of M808 for HDAC6 was 1.2 nM; the value was higher (range 466–5888 nM) for the other HDAC isoforms (Fig. 1a). The inhibitory constant (Ki) and the residence time were 8.7 nM and 48 min, respectively (Fig. 1b). These results suggest that M808 is a potent and selective HDAC6 inhibitor with a competitive binding mode of action. The viability of RA-FLS was not affected by M808 at the dose used in these experiments (Fig. 1c).

### HDAC6 inhibition increases the acetylation of tubulin and decreases the production of cytokines and MMPs by RA-FLS

**Fig. 2:**
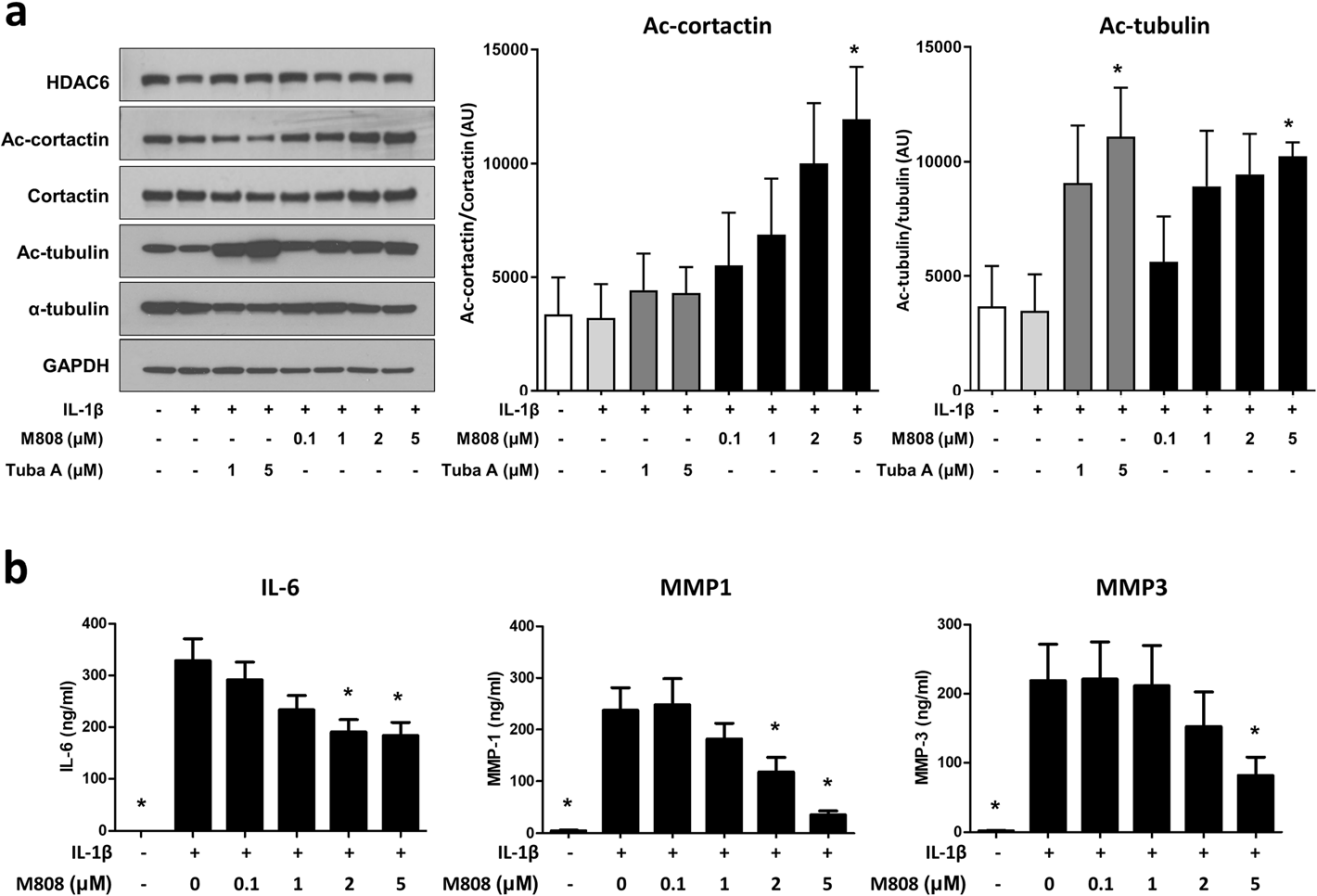
Inhibition of HDAC6 reduces the secretion of MMP-1, MMP-3, and IL-6 by fibroblast-like synoviocytes (FLS). **a** RA-FLS were activated with IL-1β (10 ng/mL) in the presence of HDAC6 inhibitor M808 and tubastatin A. The expression of HDAC6, total and acetylated cortactin, and total and acetylated α-tubulin were examined by Western blotting. A representative blot from three independent experiments is shown. Acetylated cortactin and acetylated tubulin relative to the respective total protein were shown (n = 3). Data represent the mean ± SEM. Groups were compared using a t-test. *p < 0.05 vs. FLS treated with IL-1β only. **b** FLS from RA patients (n = 6) were pre-treated for 1 h with M808 and then stimulated with IL-1β (10 ng/mL) for 24 h. The production of IL-6, MMP-1, and MMP-3 was measured in an ELISA. Data represent the mean ± SEM. Groups were compared using a t-test. *p < 0.05 vs. FLS treated with IL-1β only. AU, arbitrary unit; GAPDH, glyceraldehyde 3-phosphate dehydrogenase; IL, interleukin; MMP, metalloproteinases; Tuba A, tubastatin A

We examined whether HDAC6 inhibition influenced post-translational acetylation of the key cytoskeletal proteins tubulin and cortactin. FLS from RA patients (n = 3) were treated with IL-1β in the presence/absence of HDAC6 inhibitors. Activation of RA-FLS by IL-1β did not affect HDAC6 expression or acetylation of α-tubulin and cortactin in FLS. HDAC6 inhibition by M808 increased acetylation of α-tubulin and cortactin in a dose-dependent manner. The total amount of α-tubulin and cortactin remained stable (Fig. 2a).

Next, we examined the impact of HDAC6 inhibition on the production of IL-6, MMP-1, and MMP-3 by RA-FLS. In response to IL-1β, FLS produced large amounts of IL-6, MMP-1, and MMP3. Pretreatment with M808 decreased the production of IL-6, MMP-1, and MMP-3 in a dose-dependent manner (Fig. 2b).

### HDAC6 inhibition suppresses cell migration

**Fig. 3:**
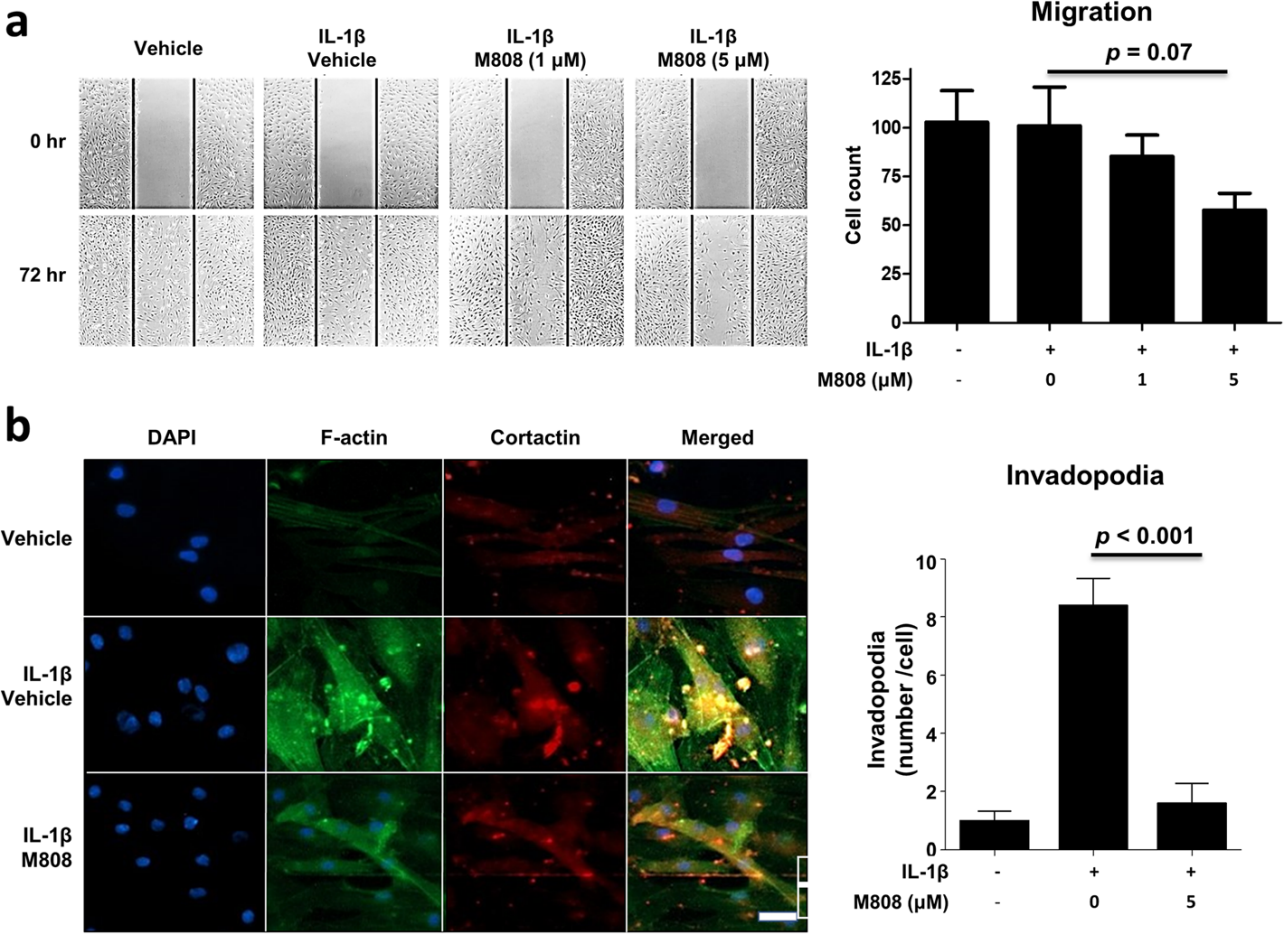
HDAC6 inhibition suppresses migration of fibroblast-like synoviocytes (FLS). **a** Cell migration assay. RA-FLS (n = 6) were treated for 1 h with M808, followed by stimulation with IL-1β for 72 h. Migration of RA-FLS in the presence of varying concentrations of M808 is depicted. Shown are representative images from six independent experiments (left panel). Original magnification × 40. The number of migrating RA-FLS in each group was compared using a t-test (right panel). **b** Following treatment with IL-1β in the presence or absence of M808 (5 μM), FLS were stained for F-actin (green) and cortactin (red). Nuclei were stained with DAPI (blue). Immunofluorescence images are representative of five independent experiments; invadopodia (yellow dots) are seen in the merged panels. Bar = 20 μm. The number of invadopodia per cell was compared between groups using a t-test (right panel). Data represented the mean ± SEM

Since cytoskeletal (re)organization is critical for cell morphology and mobility, we investigated the impact of the HDAC6 inhibition on RA-FLS migration in a cell migration assay. RA-FLS migrated and re-populated the scratched area within 72 h. In the presence of the HDAC6 inhibitor, RA-FLS migration (repopulation) tended to decrease in a dose-dependent manner (Fig. 3a).

RA-FLS morphology changed in response to IL-1β with increased cytoplasm volume and reorganization of the actin cytoskeleton; stress fibers of F-actin (phalloidin) filaments (green staining) and cortactin (red dot) increased, and more invadopodia (co-colocalization of F-actin and cortactin) were observed. M808 suppressed reorganization of F-actin and formation of invadopodia (Fig. 3b).

### HDAC6 is involved in the recruitment of immune cells

**Fig. 4:**
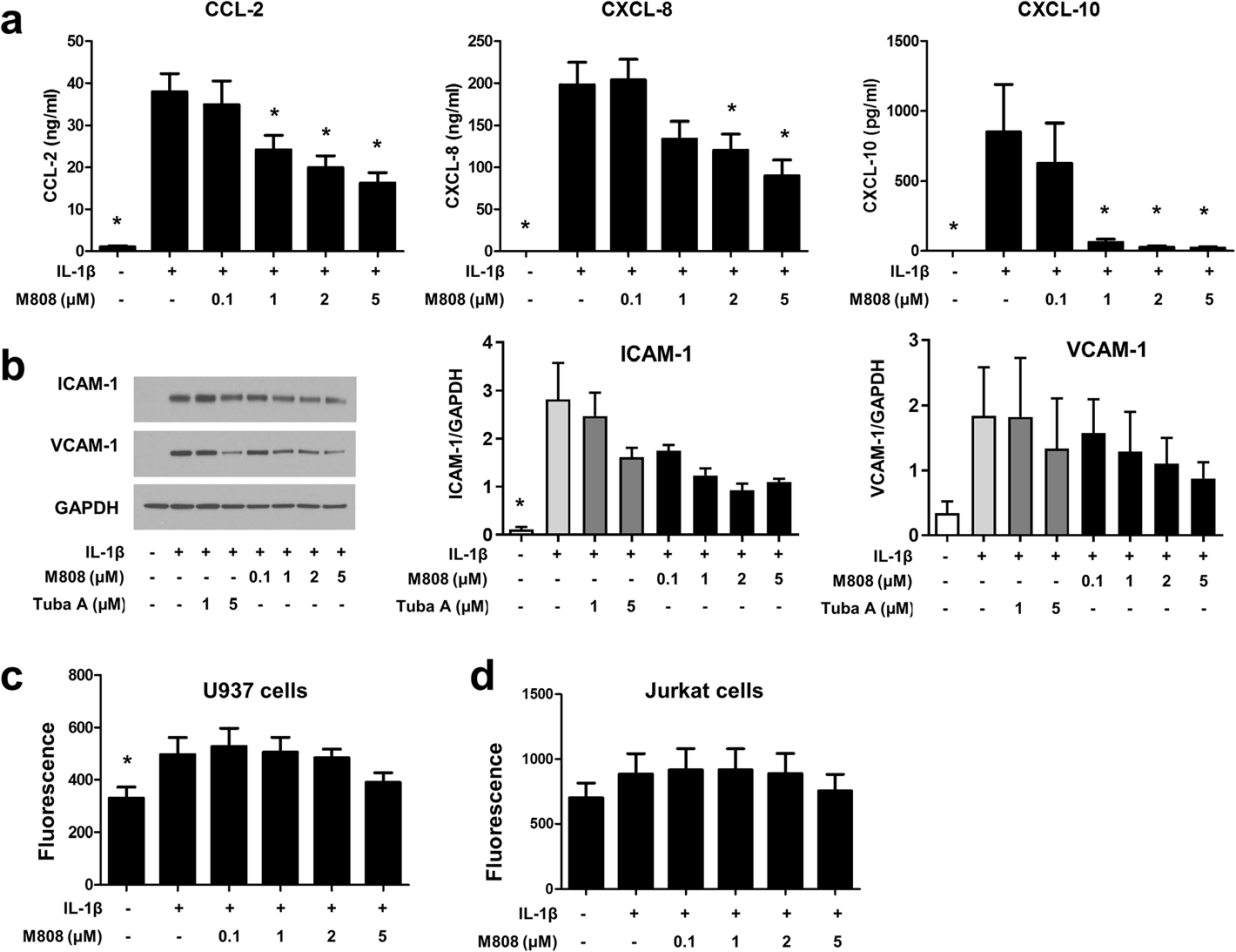
M808 inhibits chemotaxis and adhesion of inflammatory cells to RA-fibroblast-like synoviocytes (FLS). **a** RA-FLS (n = 6) were pre-treated with M808 for 1 h, followed by stimulation with IL-1β for 24 h. The amounts of CCL-2, CXCL-8, and CXCL-10 in culture supernatants were measured in an ELISA. **b** ICAM-1 and VCAM-1 expression by RA-FLS was examined by Western blotting. The image is representative of three independent experiments (left panel). The expression of ICAM-1 and VCAM-1 relative to GAPDH were quantified (middle and right panels). Data represent the mean ± SEM. **c**, **d** Adhesion of calcein-labeled U937 and Jurkat cells to RA-FLS after treatment with the HDAC6 inhibitor (n = 6). Data represent the mean ± SEM. *p < 0.05 vs. cells treated with IL-1β. GAPDH, glyceraldehyde-3-phosphate dehydrogenase; ICAM, intercellular adhesion molecule; VCAM, vascular cell adhesion molecule; Tuba, tubastatin A

Interaction between RA-FLS and immune cells is crucial for RA pathogenesis. Recruitment of immune cells into an inflammatory lesion is a critical step. Therefore, the effects of the HDAC6 inhibition on chemokine production by RA-FLS were examined. IL-1β induced RA-FLS to secrete chemokines CCL2, CXCL8, and CXCL10. In the presence of the HDAC6 inhibitor, secretion of these chemokines decreased **(**Fig. 4a).

To examine physical interaction between RA-FLS and immune cells, monocytic cell line U937 and immortalized human T cell line Jurkat cells were chosen as surrogates for monocytes and T cells, respectively. First, IL-1β treatment upregulated the expression of ICAM-1 and VCAM-1 by RA-FLS. In the presence of the HDAC6 inhibitor, the expression of both ICAM-1 and VCAM-1 tended to decrease in a dose-dependent manner (Fig. 4b). Next, RA-FLS were co-cultured with U937 or Jurkat cells in the presence of M808. HDAC6 inhibition had no significant effect on IL-1β-induced adhesion of both U937 and Jurkat cells to RA-FLS (Fig. 4c, d). Of note, IL-1β increased the adhesion of U937 cells but not that of Jurkat cells to RA-FLS.

### HDAC6 inhibition ameliorates arthritis in an AIA model

**Fig. 5:**
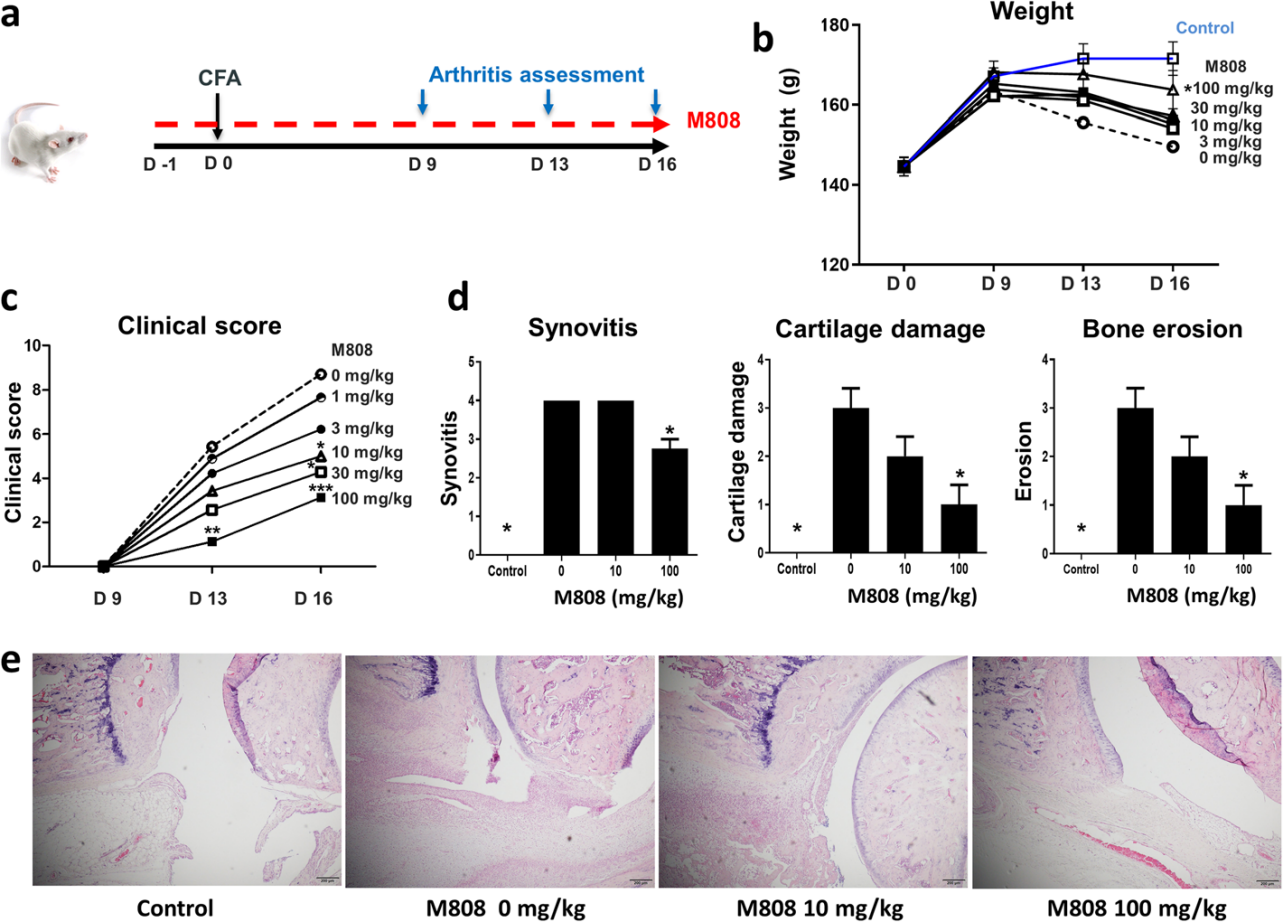
M808 increases body weight and reduces the clinical score of arthritis in an AIA model. **a** Experimental design. Arthritis was induced in Lewis rats as described in the “Methods” section. Rats received a daily dose of M808 (0, 3, 10, 30, 50, or 100 mg/kg; n = 9, 9, 9, 7, 7, and 8, respectively), starting on the day before CFA injection. **b**, **c** Weight (**b**) and clinical scores over the treatment period are shown. P values were generated using repeated measures ANOVA. **d** Synovial inflammation, bone erosion, and cartilage damage were graded after histological examination. **e** Representative histology images. Data represent the mean ± SEM. *p < 0.05, **p < 0.01, and ***p < 0.001 vs. the M808 0 mg/kg group. CFA, complete Freud’s adjuvant

To investigate the in vivo effects of HDAC6 inhibition on arthritis, Lewis rats with AIA were treated with a daily oral dose of HDAC inhibitor at different doses (Fig. 5a). In control rats, the arthritis score continued to rise until days 13 through 16. The HDAC6 inhibitor improved weight and reduced the clinical arthritis score in a dose-dependent manner (Fig. 5b, c).

Histological examination of control rats on day 16 after CFA injection revealed marked inflammation of the synovial membrane, along with cartilage destruction and bone erosion. Synovial inflammation was suppressed by the HDAC6 inhibitor. Interestingly, the HDAC6 inhibitor ameliorated joint damage (i.e., cartilage damage and bone erosion) to a greater extent than synovial inflammation (Fig. 5d, e).

## Discussion

Joint destruction in RA is mediated mainly by invasion of transformed “cancer cell-like” FLS into adjacent bones to form a pannus [3]. Here, we inhibited HDAC6 in RA-FLS and showed that subsequent PTM of the actin cytoskeleton was associated with decreased cell mobility and decreased production of tissue-degrading enzymes, inflammatory cytokines, and chemokines in vitro, and with amelioration of inflammatory arthritis in a murine model.

Since cell morphology, function, migration, and proliferation depend on constant reorganization of the cytoskeleton, PTM of the cytoskeleton, such as acetylation or deacetylation, ultimately impacts cell function and shape [24]. Therefore, regulation of HDAC6 activity, which is almost exclusively present in the cytoplasm, may be critical for normal cell physiology in immune and non-immune cells. Overexpression of HDAC6 leads to a spontaneous proinflammatory response in macrophages and a pathologically increase in HDAC6 activity in RA synovium [25, 26]. Inhibition of HDAC6 has pleiotropic effects on immune and non-immune cells; inhibiting HDAC6 promotes the generation of regulatory T cells, inhibits the proliferation of activated T cell, and suppresses inflammatory responses of activated monocytes and macrophages [20, 27, 28]. Here, we provide new evidence that HDAC6 regulates tissue-destructive RA-FLS via multiple mechanisms.

First, HDAC6 inhibition resulted in hyperacetylation of cytoskeletal proteins tubulin and cortactin in RA-FLS. This was associated with decreased production of IL-6, MMP1, and MMP3 by RA-FLS in response to IL-1β, a strong activator of FLS (Fig. 2). This is important since RA-FLS produce large amounts of tissue-degrading enzymes such as MMPs, which contribute to cartilage and bone destruction [3].

We and others reported that HDAC6 inhibition dampens the proinflammatory response by inhibiting the nuclear factor-κB (NF-κB) pathway and production of reactive oxygen species [15, 20, 26]. PTM of the cytoskeleton might be associated with altered cell signaling; indeed, acetylated microtubules amplify p38 kinase signaling and subsequent IL-10 production [29, 30]. Therefore, the production and secretion of cytokines can be regulated by modifying the cytoskeleton, which controls intracellular transport and signaling [31].

HDAC6 inhibition was associated with a decrease in cell migration (Fig. 3), which is consistent with previous studies demonstrating that acetylation of microtubules inhibits cell migration and invasion of various cell types, such as neurons and endothelial cells [32–36]. By contrast, HDAC overexpression and deacetylation of microtubules increases cell motility [17]. Tubulin acetylation increases the stability of microtubules due to increased resistance to depolymerization, leading to a rigid cytoskeletal reorganization [37]. When FLS are activated, they become larger and more confluent/adherent; they also form invadopodia, which are critical for migration and invasion along and into articular surfaces. Accordingly, M808 inhibits the morphologic transformation, including invadopodia formation, resulting in decreased cell migration (Fig. 3b) [38]. Interestingly, IL-1β treatment alone did not increase the migration of RA-FLS. It is possible that RF-FLS has already gained high mobility during a cancer-like transformation and the additional activation by IL-1β treatment might further not increase cell mobility.

A pathological hallmark of RA is the recruitment of leukocytes into synovial membranes [39]. Recruited immune cells secrete inflammatory cytokines that drive synoviocytes to transform into tissue-destructive proinflammatory RA-FLS. Here, we showed that these RA-FLS produced large amounts of chemokines. As shown previously, chemokines recruit immune cells and the infiltrating immune cells, in turn, activate FLS and together they form a positive proinflammatory feedback mechanism [5, 40]. The HDAC6 inhibitor markedly suppressed the production of chemokines CCL2, CXCL8, and CXCL10 by RA-FLS. Furthermore, it tended to downregulate the expression of adhesion molecules ICAM-1 and VCAM-1 in response to IL-1β. Adhesion via ICAM-1 and VCAM-1 might help activated RA-FLS to attach efficiently to adjacent tissues to facilitate migration/invasion. However, physical interactions between FLS and immune cells (U937 and Jurkat as surrogates for monocytes and T cells, respectively) did not decrease significantly upon HDAC6 inhibition, suggesting that adherence of immune cells to RA-FLS is significantly influenced by HDAC6 inhibition (Fig. 4).

Finally, HDAC6 inhibitor M808 ameliorated clinical arthritis in an AIA-murine arthritis model in a dose-dependent manner (Fig. 5). These findings are consistent with the anti-inflammatory and joint-protective effects of other HDAC6 inhibitors [19, 20]. It is striking that HDAC6 inhibition suppressed bone and cartilage damage more than synovial inflammation, emphasizing the inhibitory effect of HDAC6 on activated FLS during synovial inflammation. Taken together, the data suggest that HDAC6 plays an important role in the tissue-destructive function of RA-FLS during RA pathogenesis and that HDAC6 inhibition might have therapeutic potential in patients with RA.

Further studies are needed to investigate the mechanisms (i.e., hyper- and deacetylation of cytoskeletal proteins such as tubulin impacts) by which HDAC6 (or its inhibition) affect cytoskeletal (re)organization and subsequent cellular functions, including expression of cytokines, chemokines, and adhesion molecules. Cells that require rapid cytoskeletal reorganization, such as activated or proliferating cells, might be more susceptible to HDAC6 inhibitors.

## Conclusion

In conclusion, M808, a novel specific HDAC6 inhibitor, suppresses RA-FLS-mediated inflammatory responses and tissue destruction in a murine RA model. Further studies are needed to investigate whether HDAC6 inhibition has therapeutic effects in patients with RA.

## Data Availability

The datasets generated during and/or analyzed during the current study are available from the corresponding author on reasonable request.

## Abbreviations

ACR: American College of Rheumatology
AIA: Adjuvant-induced arthritis
CFA: Complete Freund’s adjuvant
ELISA: Enzyme-linked immunosorbent assay
FLS: Fibroblast-like synoviocytes
HDAC: Histone deacetylase
IL: Interleukin
MMP: Metalloproteinase
NF-κB: Nuclear factor-κB
PBS: Phosphate-buffered saline
PTM: Post-translational modification
RA: Rheumatoid arthritis
TNF: Tumor necrosis factor

## References

[1] Firestein GS (2003). Evolving concepts of rheumatoid arthritis. Nature.

[2] McInnes IB, Schett G (2017). Pathogenetic insights from the treatment of rheumatoid arthritis. Lancet.

[3] Bartok B, Firestein GS (2010). Fibroblast-like synoviocytes: key effector cells in rheumatoid arthritis. Immunol Rev.

[4] Lee DM, Kiener HP, Agarwal SK, Noss EH, Watts GF, Chisaka O, Takeichi M, Brenner MB (2007). Cadherin-11 in synovial lining formation and pathology in arthritis. Science.

[5] Smolen JS, Steiner G (2003). Therapeutic strategies for rheumatoid arthritis. Nat Rev Drug Discov.

[6] Grabiec AM, Reedquist KA (2013). The ascent of acetylation in the epigenetics of rheumatoid arthritis. Nat Rev Rheumatol.

[7] Grabiec AM, Korchynskyi O, Tak PP, Reedquist KA (2012). Histone deacetylase inhibitors suppress rheumatoid arthritis fibroblast-like synoviocyte and macrophage IL-6 production by accelerating mRNA decay. Ann Rheum Dis.

[8] Gillespie J, Savic S, Wong C, Hempshall A, Inman M, Emery P, Grigg R, McDermott MF (2012). Histone deacetylases are dysregulated in rheumatoid arthritis and a novel histone deacetylase 3-selective inhibitor reduces interleukin-6 production by peripheral blood mononuclear cells from rheumatoid arthritis patients. Arthritis Rheum.

[9] Gregoretti IV, Lee YM, Goodson HV (2004). Molecular evolution of the histone deacetylase family: functional implications of phylogenetic analysis. J Mol Biol.

[10] Kim DH, Kim M, Kwon HJ (2003). Histone deacetylase in carcinogenesis and its inhibitors as anti-cancer agents. J Biochem Mol Biol.

[11] Lee JH, Mahendran A, Yao Y, Ngo L, Venta-Perez G, Choy ML, Kim N, Ham WS, Breslow R, Marks PA (2013). Development of a histone deacetylase 6 inhibitor and its biological effects. Proc Natl Acad Sci U S A.

[12] Dallavalle S, Pisano C, Zunino F (2012). Development and therapeutic impact of HDAC6-selective inhibitors. Biochem Pharmacol.

[13] Valenzuela-Fernandez A, Cabrero JR, Serrador JM, Sanchez-Madrid F (2008). HDAC6: a key regulator of cytoskeleton, cell migration and cell-cell interactions. Trends Cell Biol.

[14] Kato M, Ospelt C, Kolling C, Shimizu T, Kono M, Yasuda S, Michel BA, Gay RE, Gay S, Klein K, Atsumi T (2016). AAA-ATPase p97 suppresses apoptotic and autophagy-associated cell death in rheumatoid arthritis synovial fibroblasts. Oncotarget.

[15] Soo Youn G, Ju SM, Choi SY, Park J. HDAC6 mediates HIV-1 tat-induced proinflammatory responses by regulating MAPK-NF-kappaB/AP-1 pathways in astrocytes. Glia. 2015. 10.1002/glia.22865 26031809

[16] Lee YS, Lim KH, Guo X, Kawaguchi Y, Gao Y, Barrientos T, Ordentlich P, Wang XF, Counter CM, Yao TP (2008). The cytoplasmic deacetylase HDAC6 is required for efficient oncogenic tumorigenesis. Cancer Res.

[17] Hubbert C, Guardiola A, Shao R, Kawaguchi Y, Ito A, Nixon A, Yoshida M, Wang XF, Yao TP (2002). HDAC6 is a microtubule-associated deacetylase. Nature.

[18] Zhang X, Yuan Z, Zhang Y, Yong S, Salas-Burgos A, Koomen J, Olashaw N, Parsons JT, Yang XJ, Dent SR, Yao TP, Lane WS, Seto E (2007). HDAC6 modulates cell motility by altering the acetylation level of cortactin. Mol Cell.

[19] Vishwakarma S, Iyer LR, Muley M, Singh PK, Shastry A, Saxena A, Kulathingal J, Vijaykanth G, Raghul J, Rajesh N, Rathinasamy S, Kachhadia V, Kilambi N, Rajgopal S, Balasubramanian G, Narayanan S (2013). Tubastatin, a selective histone deacetylase 6 inhibitor shows anti-inflammatory and anti-rheumatic effects. Int Immunopharmacol.

[20] Park JK, Jang YJ, Oh BR, Shin J, Bae D, Ha N, Choi YI, Youn GS, Park J, Lee EY (2020). Therapeutic potential of CKD-506, a novel selective histone deacetylase 6 inhibitor, in a murine model of rheumatoid arthritis. Arthritis Res Ther.

[21] Santo L, Hideshima T, Kung AL, Tseng JC, Tamang D, Yang M, Jarpe M, van Duzer JH, Mazitschek R, Ogier WC, Cirstea D, Rodig S, Eda H, Scullen T, Canavese M, Bradner J, Anderson KC, Jones SS, Raje N (2012). Preclinical activity, pharmacodynamic, and pharmacokinetic properties of a selective HDAC6 inhibitor, ACY-1215, in combination with bortezomib in multiple myeloma. Blood.

[22] Wegener D, Wirsching F, Riester D, Schwienhorst A (2003). A fluorogenic histone deacetylase assay well suited for high-throughput activity screening. Chem Biol.

[23] Arnett FC, Edworthy SM, Bloch DA, McShane DJ, Fries JF, Cooper NS, Healey LA, Kaplan SR, Liang MH, Luthra HS (1988). The American Rheumatism Association 1987 revised criteria for the classification of rheumatoid arthritis. Arthritis Rheum.

[24] Yang XJ, Seto E (2007). HATs and HDACs: from structure, function and regulation to novel strategies for therapy and prevention. Oncogene.

[25] Horiuchi M, Morinobu A, Chin T, Sakai Y, Kurosaka M, Kumagai S (2009). Expression and function of histone deacetylases in rheumatoid arthritis synovial fibroblasts. J Rheumatol.

[26] Youn GS, Lee KW, Choi SY, Park J (2016). Overexpression of HDAC6 induces pro-inflammatory responses by regulating ROS-MAPK-NF-kappaB/AP-1 signaling pathways in macrophages. Free Radic Biol Med.

[27] Oh BR, Suh DH, Bae D, Ha N, Choi YI, Yoo HJ, Park JK, Lee EY, Lee EB, Song YW (2017). Therapeutic effect of a novel histone deacetylase 6 inhibitor, CKD-L, on collagen-induced arthritis in vivo and regulatory T cells in rheumatoid arthritis in vitro. Arthritis Res Ther.

[28] Cantley MD, Bartold PM, Fairlie DP, Rainsford KD, Haynes DR (2012). Histone deacetylase inhibitors as suppressors of bone destruction in inflammatory diseases. J Pharm Pharmacol.

[29] Wang B, Rao YH, Inoue M, Hao R, Lai CH, Chen D, McDonald SL, Choi MC, Wang Q, Shinohara ML (2014). Microtubule acetylation amplifies p38 kinase signalling and anti-inflammatory IL-10 production. Nat Commun.

[30] Janke C, Montagnac G (2017). Causes and consequences of microtubule acetylation. Curr Biol.

[31] Boyault C, Sadoul K, Pabion M, Khochbin S (2007). HDAC6, at the crossroads between cytoskeleton and cell signaling by acetylation and ubiquitination. Oncogene.

[32] Creppe C, Malinouskaya L, Volvert ML, Gillard M, Close P, Malaise O, Laguesse S, Cornez I, Rahmouni S, Ormenese S, Belachew S, Malgrange B, Chapelle JP, Siebenlist U, Moonen G, Chariot A, Nguyen L (2009). Elongator controls the migration and differentiation of cortical neurons through acetylation of alpha-tubulin. Cell.

[33] Deakin NO, Turner CE (2014). Paxillin inhibits HDAC6 to regulate microtubule acetylation, Golgi structure, and polarized migration. J Cell Biol.

[34] Wang YH, Yan ZQ, Qi YX, Cheng BB, Wang XD, Zhao D, Shen BR, Jiang ZL (2010). Normal shear stress and vascular smooth muscle cells modulate migration of endothelial cells through histone deacetylase 6 activation and tubulin acetylation. Ann Biomed Eng.

[35] Kaluza D, Kroll J, Gesierich S, Yao TP, Boon RA, Hergenreider E, Tjwa M, Rossig L, Seto E, Augustin HG (2011). Class IIb HDAC6 regulates endothelial cell migration and angiogenesis by deacetylation of cortactin. EMBO J.

[36] Zhang Y, Zhang M, Dong H, Yong S, Li X, Olashaw N, Kruk PA, Cheng JQ, Bai W, Chen J, Nicosia SV, Zhang X (2009). Deacetylation of cortactin by SIRT1 promotes cell migration. Oncogene.

[37] Li L, Yang XJ (2015). Tubulin acetylation: responsible enzymes, biological functions and human diseases. Cell Mol Life Sci.

[38] Muller-Ladner U, Kriegsmann J, Franklin BN, Matsumoto S, Geiler T, Gay RE, Gay S (1996). Synovial fibroblasts of patients with rheumatoid arthritis attach to and invade normal human cartilage when engrafted into SCID mice. Am J Pathol.

[39] Komatsu N, Takayanagi H (2012). Inflammation and bone destruction in arthritis: synergistic activity of immune and mesenchymal cells in joints. Front Immunol.

[40] Palmer G, Gabay C, Imhof BA (2006). Leukocyte migration to rheumatoid joints: enzymes take over. Arthritis Rheum.

